# Supplementary material for: Gut-derived metabolite 3-methylxanthine enhances cisplatin-induced apoptosis via dopamine receptor D1 in a mouse model of ovarian cancer
Source: mSystems. 2024 Jun 20;9(7):e01301-23. doi: 10.1128/msystems.01301-23 (PMC11264688; doi:10.1128/msystems.01301-23)
Supplement: Supplemental Figures — Figures S1-S5. [file msystems.01301-23-s0001.pdf]

This file includes:

Supplementary figures S1-S5

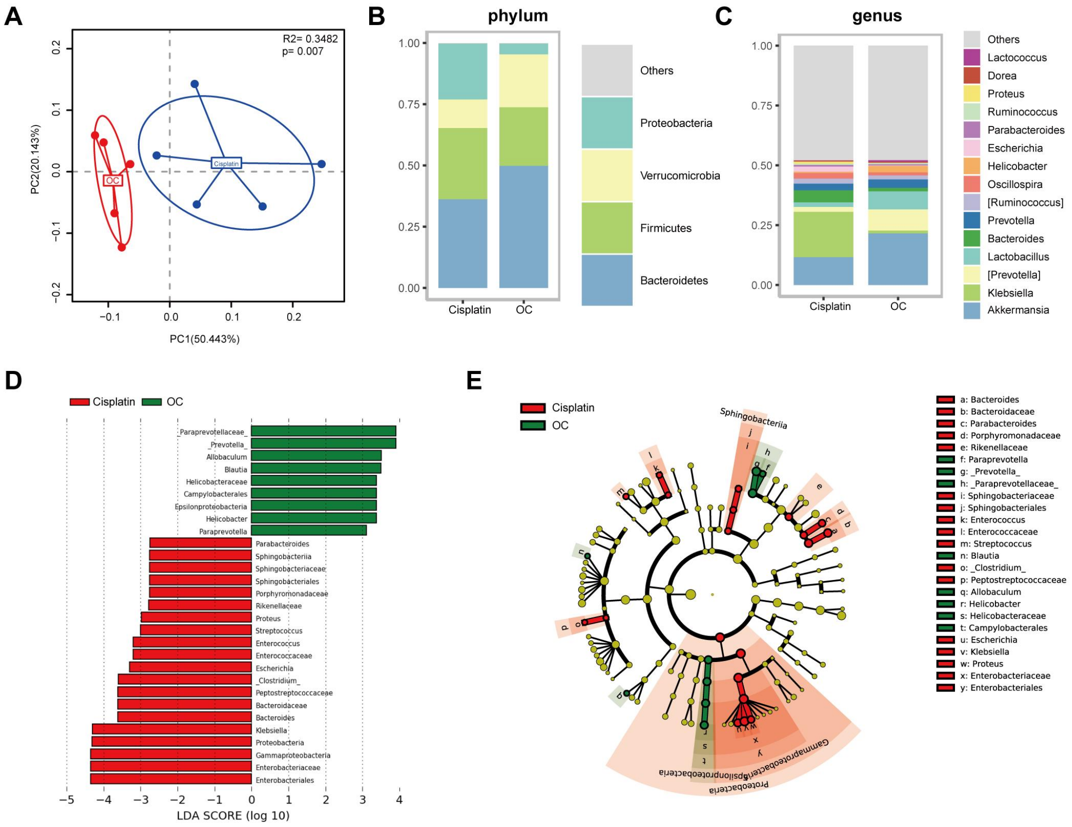

Fig. S1 Cisplatin treatment altered the composition of gut microbiota in OC-bearing mice.

(A) Principal coordinate analysis (PCoA) based on the Binary Jaccard distance analysis of operational taxonomic units (OTUs).

(B)(C) Relative abundance at the phylum and genus level between OC and cisplatin groups.

(E)(F) LDA along with effect size measurements was applied to present the enriched bacteria in each group in cecum content.

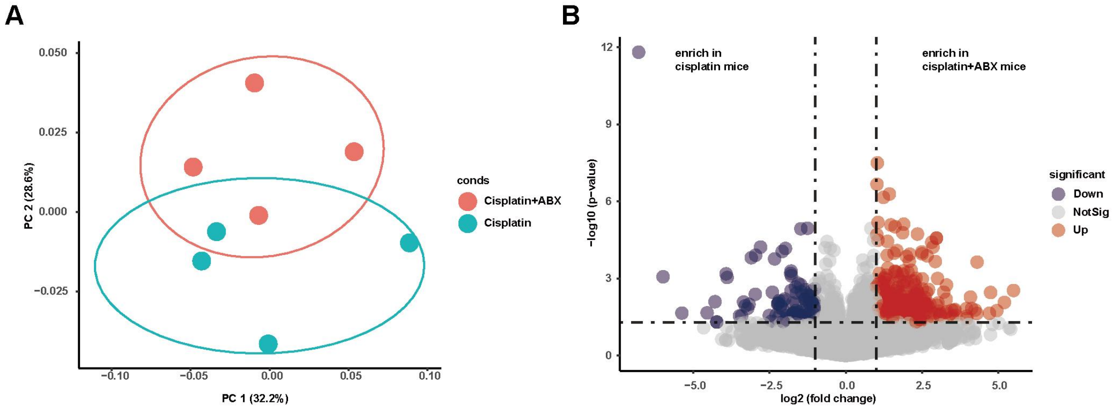

Fig. S2 Administration of antibiotics resulted in modifications to the transcriptional profile of tumors in mice with OC.

(A) Principal component analysis (PCA) of RNA composition between cisplatin and cisplatin+ABX treated mice (n=4).

(B) Volcano plot analysis reveals that 377 genes upregulated in cisplatin+ABX treated mice.

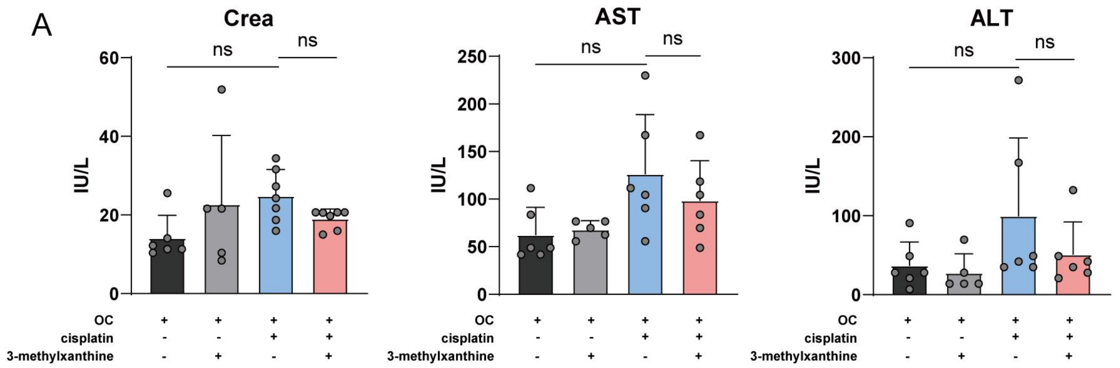

Fig. S3 3-methylxanthine treatment was well tolerated without any discernible toxicological effects.

(A) Plasma Crea, ALT and AST levels at the endpoint. Data are presented as the mean ± SEM, ns, no significant difference.

A

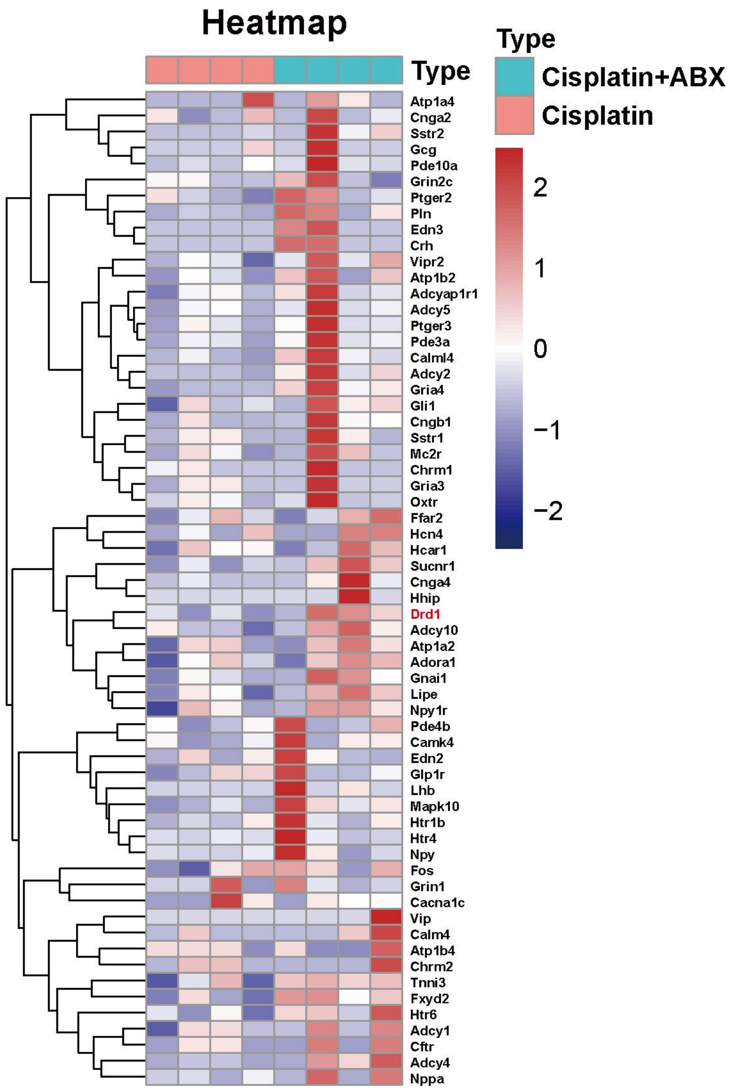

Fig. S4 Antibiotic treatment induced alterations in the expression of genes related to the cAMP signaling pathway in tumors of mice with OC.

(A) Heatmap shows the expression of cAMP signaling pathway related genes.

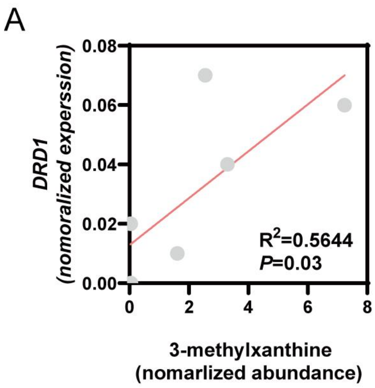

Fig. S5 A positive correlation was observed between 3-methylxanthine and DRD1.

(A) Correlation between 3-methlyxanthine abundance in cecum content and DRD1 expression in tumor of OC-bearing mice.
